# German-language adaptation of the Bereavement Risk Assessment Tool (BRAT): findings on the inter-rater reliability

**DOI:** 10.1186/s12904-026-02033-z

**Published:** 2026-03-02

**Authors:** Kristina Klug, Heidi Müller, Stephan Bongard, Ulf Sibelius, Bastian Eul, Tatjana Huys, Daniel Berthold

**Affiliations:** 1 Department of Psychology, Individual Differences & Psychological Assessment, Goethe University, Theodor-W.-Adorno-Platz 6, Frankfurt Am Main, 60323 Germany; 2 Department of Internal Medicine, Palliative Care, Faculty of Medicine, University Hospital Giessen and Marburg (UKGM), Justus Liebig University Giessen, Giessen Campus, Giessen, Germany; 3 Department of Internal Medicine, Universities of Giessen and Marburg Lung Center (UGMLC), Justus-Liebig-University, Giessen, Germany; 4 German Center for Lung Research (DZL), Giessen, Germany

**Keywords:** Bereavement, Risk assessment, Prolonged grief disorder, Inter-rater reliability, Palliative care, Cross-cultural validation, Healthcare professionals

## Abstract

**Background:**

Prolonged Grief Disorder (PGD) affects approximately 10% of the bereaved adults, with prevalence rates in Germany ranging between 4.7–5.4%. Early identification through pre-death risk assessment is crucial for optimal bereavement care, yet no validated German-language instrument exists. The Bereavement Risk Assessment Tool (BRAT) represents a promising solution but requires cultural adaptation and validation. The aim of this study was to adapt the BRAT for German-speaking countries and evaluate its inter-rater reliability among healthcare professionals in palliative care and hospice settings.

**Methods:**

A cross-sectional reliability study was conducted online. The BRAT was translated into German using forward and backward translation methodology, resulting in the Multiprofessioneller Fragebogen zur Trauerverarbeitung (MFT). Participants evaluated four case vignettes featuring 10 bereaved family members using the 40-item MFT. Inter-rater reliability was assessed using Gwet's AC and Krippendorff's α across multiple risk categorizations.

**Results:**

A total of 55 healthcare professionals (78.2% female,* M* = 51.5 years) working in German palliative care and hospice settings were recruited. Participants included diverse professional groups: nursing staff, pastoral care workers, medical doctors, psychologists, and social workers. The MFT demonstrated good to excellent inter-rater reliability across most items. The majority of items showed good to almost perfect agreement between raters. Notable variations emerged between professional groups, with inter-rater reliability being influenced by the number of response categories used for risk assessment.

**Conclusions:**

The German adaptation represents a reliable tool for pre-death bereavement risk assessment in German-speaking healthcare settings. Profession-specific training programs may enhance consistency across occupational groups. Further validation studies examining predictive validity for PGD outcomes are warranted.

## Introduction

Since 2021, more than 1 million people have died each year in Germany [1]. Estimates suggest that each deceased person leaves behind several close relatives and members of the wider social network, commonly ranging from two to five individuals [2–4]. Even when adopting conservative assumptions, this implies that several million people in Germany are affected by the death of a loved one each year.

Grieving individuals are at heightened risk of experiencing a variety of life-debilitating mental and physical health conditions [5]. However, the majority of people affected manage to cope with the loss of a loved one over time, supported by their social networks and communities [2]. Nevertheless, grief can also take on forms that severely impair the quality of life of those affected [6]. Meaning for example that long after the death of their loved one people are often still not able to cope with everyday life. This condition, characterised by elevated and persistent mental distress following the loss, has been formally recognised as Prolonged Grief Disorder (PGD) in both the International Classification of Diseases, 11th Edition (ICD-11; [7]) and the Diagnostic and Statistical Manual of Mental Disorders, 5th Edition, Text Revision (DSM-5-TR; [8]). PGD is characterized by intense yearning or preoccupation, emotional distress, and other symptoms that should be diagnosed no earlier than six months (according to the ICD-11) or 12 months (according to the DSM-5-TR) after the loss. The diagnosis acknowledges the distinct nature of pathological grief reactions and represents a significant milestone in grief research and clinical practice [6]. In this manuscript, the term *prolonged grief disorder (PGD)* is used when referring to the formally recognized diagnostic entity as defined in ICD-11 and DSM-5-TR. The broader terms *prolonged grief* or *prolonged grief reactions* are used descriptively to refer to persistent grief-related symptoms that may or may not meet full diagnostic criteria. This distinction is made to reflect both clinical and subclinical manifestations discussed in the literature.

Studies more or less carefully indicate that approximately around 10% of the bereaved adults experience the persistent symptoms of prolonged grief disorder [9], with prevalence rates in Germany ranging between 4,7% (according DSM-5-TR criteria) and 5,4% (according ICD-11 criteria) of the bereaved experience this prolonged debilitating grief reactions [10]. The aim was to help identify people affected by PGD, so that they can be offered the best available intervention [11]. The global scope of this challenge has become even more evident during the COVID-19 pandemic, which has created unprecedented circumstances for grief and bereavement, with millions of people worldwide experiencing loss under conditions that seem to have increased the risk for prolonged grief reactions [12].

Unrecognised prolonged grief is not only associated with poor health prognosis but it has as well significant economic implications for individuals, families, and societies. For example, in Scotland, spousal bereavement has been correlated with earlier death and more hospital consultations and stays, adding costs of £20 million annually [13]. Additionally, medical costs of bereavement are further compounded by bereavement-related reductions in productivity and unemployment. For example, bereavement is associated with sick leave [14] and income loss [15]. Recent evidence suggests that bereavement should be considered an important public health issue, yet economic and resource investments to effectively implement and sustain integrated bereavement services are sorely lacking at national and global levels [16]. Thus, from both a therapeutic and a health policy perspective, structured identification of individuals at increased risk to develop difficulties in their grieving processes may support targeted allocation of bereavement care resources.

In numerous studies risk factors for developing prolonged grief reactions have been identified [17, 18]. These relate to a) personal factors such as gender, low income, older age; b) interpersonal factors such as closeness to the deceased, availability of support; c) situational factors such as cause of death and d) societal and cultural factors such as existing norms related to grief and prolonged grief reactions [19]. Although they have received less attention than risk factors, studies have also identified protective factors such as religion and social support [20, 21]. Bereavement risk screening has repeatedly been highlighted as an important component of optimal care [22–24], yet it has not been consistently implemented by palliative care or oncology services [25, 26]. Given that higher risk individuals often report the most unmet need [2, 27], bereavement resources might be best allocated to those who are most distressed.

According to the WHO palliative care not only improves the well-being of the dying but as well the quality of life of caregivers [28]. While some institutions and practices may offer bereavement services such as bereavement groups or individual counselling, there is significant variability in the availability of services and outreach, with many services delivered in an ad hoc basis without formal assessment of risk or need [29]. This is particularly true in Germany, where palliative and bereavement care have developed independently of each other and bereavement care varies widely in terms of e.g., availability, diagnostics and quality of interventions [30, 31]. As a result, those who struggle the most tend to face barriers to accessing care, often “falling through the cracks” [29, 32, 33]. The COVID-19 pandemic has further highlighted these gaps, with studies showing that healthcare systems worldwide struggled to provide adequate bereavement support during a time of unprecedented need [34]. In this context, identifying those most in need of support can facilitate more effective care coordination [22, 24].

### Early recognition of prolonged grief

The palliative care and hospice sector is a healthcare area in which dying, death, farewell and grief naturally become a topic and a challenge for relatives (e.g., [35–37]). Risk assessment for the possible development of prolonged grief reactions is considered a crucial first step in determining the need for grief support and targeting it to those who need it most [24, 38]. Recent systematic reviews have emphasised that effective bereavement risk assessment is essential for implementing evidence-based, tiered models of bereavement care [39]. For many years, professional literature has repeatedly emphasised the need for reliable and valid assessment tools, as these are seen as an essential part of a wider commitment to standards in bereavement, palliative and hospice care as a whole (e.g., [29, 40, 41]). Contemporary assessment approaches must now align with the new ICD-11 and DSM-5-TR criteria for prolonged grief disorder, requiring updated validation of existing instruments [42, 43]. In the palliative and hospice sector, post-death assessment appears to be difficult as professionals rarely have face-to-face contact with families after death, so pre-death risk assessment tools may be more appropriate in this sector [29]. Reviews on pre-death screening tools [24, 44] revealed two available tools suitable for the palliative and hospice sector: the Bereavement Risk Index (BRI; [45, 46]) and the Bereavement Risk Assessment Tool (BRAT; [47]). Compared to the BRI, the BRAT offers two key advantages: it includes protective factors and provides a more comprehensive assessment of potential stressors, such as lack of social support and concurrent stressors. The other three pre-death assessment tools found focus on relatives of patients with dementia and do not cover the full spectrum of palliative conditions.

In the German-speaking world, however, no validated pre-death instrument exists to date to determine the risk of developing difficulties in the grieving process [44]. This gap is particularly concerning given the new diagnostic criteria for prolonged grief disorder and the need for culturally adapted assessment tools that can function effectively within the German healthcare system [10]. The aim of this study is therefore to fully transfer a pre-death risk assessment tool to German-speaking countries and regions, including testing the instrument for inter-rater reliability using different professional groups from the palliative care and hospice sector.

Based on these considerations, the Bereavement Risk Assessment Tool (BRAT) was selected for cultural adaptation and evaluation in German-speaking palliative care settings. It is a risk assessment tool developed by the Victoria Hospice Society in Canada [47]. It aims at determining difficulties in the grief processes for each relative using a two-page questionnaire. To date, the BRAT is considered a working version that can be used in practice. However, it has not yet undergone formal psychometric validation in the German language.

## Method

### Tool

The Bereavement Risk Assessment Tool (BRAT; [48]) consists of 40 items, including 36 risk factors (e.g., mental challenges, previous losses) and 4 protective factors (e.g., spiritual and religious beliefs), designed to collectively predict the likelihood of complications or difficulties during bereavement. It was developed by a multidisciplinary team of palliative care and hospice professionals. Drawing on clinical expertise, each factor was assigned a numerical value corresponding to one of five risk levels, indicating varying degrees of relative risk. The questionnaire is filled in gradually over time by the multi-professional team in order to obtain as complete a picture as possible of a patient's relatives. The weighted 40 items are calculated into a cumulative score, which is divided into five risk levels: “no known risk” (level 1), “minimal risk” (level 2), “low risk” (level 3), “moderate risk” (level 4), and “high risk” (level 5). A recommendation for a different intensity of therapeutic intervention is formulated for each risk level. Following the death there is no formal questioning of the bereaved family member. In July 2014, we obtained written authorisation from the Victoria Hospice Society to use and adapt the tool to the German language. Thus, the original BRAT was translated into German using a standardized forward–backward translation procedure. This process consisted of a cyclical approach, i.e. translation into German, back-translation into English and a comparative review of the back-translation with the original until a consensus on the translation was reached between the two experts. The translators were native speakers of the language into which they were translating and be familiar with working in the palliative care sector. Discrepancies between the original and back-translated versions were reviewed and resolved through consensus discussions among the translators and two of the authors (H.M., D.B.), which led to the final German version. This approach is widely recommended for cross-cultural adaptation of assessment instruments and has been shown to enhance semantic, conceptual, and cultural equivalence as well as inter-rater reliability [49, 50]. The German version of the BRAT was named Multiprofessioneller Fragebogen zur Trauerverarbeitung (MFT).

### Design

The study was funded by the Deutsche PalliativStiftung (DPS, translated: German Palliative Care Foundation) and approved by the institutional research ethics committee at the Faculty of Medicine, Justus Liebig University Giessen (AZ 158/15). The study was designed as an online study using the online survey tool SosciSurvey. The study design involved asking participants to review four case studies, each featuring 10 family members and caregivers of palliative care patients. These case studies were originally developed as part of the BRAT by four experienced members of a Canadian clinical team, who had significant expertise in using the BRAT. As in the original study, the raters were provided with the BRAT’s indicator descriptors. All cases and indicator descriptors were translated via forward and back translation method into German in a similarly way to the BRAT. Participants for the study in Germany were recruited via snowball method and targeted outreach from among employees from all occupational groups, all of whom reported working at least part-time in the palliative care or hospice sector or in a closely related field. Snowball sampling was chosen because the target population represents a specialized professional group that is difficult to reach through probabilistic sampling methods. This approach facilitated access to experienced professionals across multiple disciplines and institutions and allowed for efficient recruitment across professional boundaries. Cluster sampling was considered but not applied due to feasibility constraints and the exploratory focus of the study. Implementing cluster sampling would have required systematic access to a large number of institutions and formal organizational participation, which was beyond the scope of this initial inter-rater reliability study. Given that the primary aim was to examine agreement between raters rather than to derive population-level estimates, this recruitment strategy was considered appropriate for the study objectives. Participants were informed about data protection, anonymity, and adherence to the ethical guidelines of the German Psychological Society (DGPs) and the Professional Association of German Psychologists (BDP) in the invitation email. Completion of the survey was taken to indicate informed consent.

### Statistical analysis

Reliability analyses were conducted, including intra-class correlations to assess the absolute agreement of MFT-generated risk levels [51]. Inter-rater reliability for individual items on the tool as well as the overall MFT risk levels, was evaluated using Gwet’s *AC* or Krippendorffs α for ordinal data instead of Fleiss’ kappa since Cicchetti and Feinstein [52] described the paradox of Fleiss’ kappa where high agreement is coupled with low κ. It is therefore an important test requirement that the characteristics (selected/not selected) are distributed more or less equally between the subjects (relatives). Thus, we decided to use Gwet's *AC*, which is specially programmed for heterogeneous distributions [53, 54]

## Results

### Respondents

The assessment tool was completed by a total of 55 participants (78.2% female, 21.8% male), who were on average 51.5 years old (*SD* = 10.9, range 29–75). 25.5% (*n* = 14) of these individuals worked in the nursing sector, 20.0% (*n* = 11) in pastoral care, and 18.2% (*n* = 10) each in medicine, psychology, and social work. Of the 55 participants, approximately one third (34.5%, *n* = 19) had less than 5 years of professional experience, 29.1% (*n* = 16) stated to have 5–10 years of professional experience in the palliative care or hospice sector, 18.2% (*n* = 10) stated they have 10–15 years of professional experience, and 18.2% (*n* = 10) stated they have more than 15 years of professional experience. Only 7 of the 55 people stated to already have experience with diagnostic instruments in the palliative care or hospice sector.

### Inter-rater reliability

For the 40 MFT items used in this study, values of Gwet’s *AC* ranged from 0.399 to 0.996 (Table 1). Only one item (Item 25) had a Gwet’s *AC* value less than 0.4 (slight to fair agreement), 7 items had Gwet’s *AC* values less than 0.7 (moderate to good agreement), and the remaining 32 items (80%) had Gwet’s *AC* values above 0.7 (good to almost perfect agreement) [55].

**Table 1:** Gwet’s AC, standard error, t-values, *p*-values, and 95% CI for the 40 MFT items

Item No	Gwet’s *AC*	Std.Err	*t*	*p*	[95%Conf	Interval]
1	0.969	0.013	71.810	0.000	0.938	0.999
2	0.793	0.071	11.090	0.000	0.631	0.955
3	0.469	0.107	4.380	0.002	0.227	0.711
4	0.969	0.012	79.580	0.000	0.941	0.996
5	0.996	0.004	72.010	0.000	0.988	1.000
6	0.944	0.052	18.220	0.000	0.827	1.000
7	0.947	0.038	24.710	0.000	0.860	1.000
8	0.989	0.011	92.150	0.000	0.965	1.000
9	0.770	0.126	6.120	0.000	0.485	1.000
10	0.633	0.159	3.990	0.003	0.274	0.992
11	0.938	0.025	37.520	0.000	0.881	0.995
12	0.892	0.066	13.560	0.000	0.743	1.000
13	0.939	0.037	25.370	0.000	0.855	1.000
14	0.961	0.024	40.940	0.000	0.908	1.000
15	0.824	0.090	9.150	0.000	0.620	1.000
16	0.838	0.096	8.740	0.000	0.621	1.000
17	0.770	0.109	7.050	0.000	0.523	1.000
18	0.852	0.098	8.700	0.000	0.630	1.000
19	0.712	0.137	5.200	0.001	0.402	1.000
20	0.890	0.061	14.650	0.000	0.753	1.000
21	0.825	0.110	7.490	0.000	0.576	1.000
22	0.972	0.029	34.130	0.000	0.907	1.000
23	0.753	0.136	5.540	0.000	0.446	1.000
24	0.971	0.025	38.540	0.000	0.914	1.000
25	0.399	0.127	3.140	0.012	0.112	0.687
26	0.878	0.073	12.010	0.000	0.713	1.000
27	0.978	0.011	87.030	0.000	0.953	1.000
28	0.989	0.008	126.630	0.000	0.971	1.000
29	1.000	-	-	-	-	-
30	0.978	0.013	78.150	0.000	0.950	1.000
31	0.978	0.011	85.900	0.000	0.952	1.000
32	0.920	0.041	22.390	0.000	0.827	1.000
33	0.654	0.131	4.990	0.001	0.358	0.951
34	0.881	0.058	15.220	0.000	0.750	1.000
35	0.891	0.078	11.400	0.000	0.714	1.000
36	0.915	0.059	15.630	0.000	0.783	1.000
37	0.480	0.155	3.090	0.013	0.129	0.831
38	0.470	0.129	3.630	0.005	0.177	0.763
39	0.599	0.160	3.760	0.005	0.238	0.960
40	0.682	0.137	4.980	0.001	0.372	0.992

Items 27–30 refer exclusively to application to relatives under 18 years of age

To examine the inter-rater reliability across different occupational groups from the palliative care and hospice sector we first analysed the original responses categorised on a five-point ordinal scale and then summarised the responses in three stages as “high versus medium versus low risk” and then in two stages as “high versus low risk”. To investigate the inter-rater reliability Krippendorff's α for ordinal data was used [56]. The analyses were carried out using the KALPHA module for IBM SPSS Statistics Version 26.

#### Inter-rater reliability across the five-level response categories

Across all 55 raters, Krippendorff's α was 0.580 (95% CI 0.559–0.589). A specific analysis of the occupational groups can be found in Table 2.

**Table 2:** Krippendorff's α for different occupational groups across the five-level response categories

Occupation	Krippendorff ‘s α	95% CI
Nursing (*n* = 14)	0.628	0.592–0.662
Medicine (*n* = 10)	0.654	0.605–0.669
Psychology (*n* = 10)	0.543	0.482–0.603
Pastoral care (*n* = 11)	0.485	0.428–0.539
Social work (*n* = 10)	0.542	0.478–0.601

#### Inter-rater reliability across the three-level response categories

The data was categorised on three levels: 1 = *No known or minimal risk*, 2 = *Low risk*, 3 = *Moderate or high risk*. Across all 55 raters, Krippendorff's α was 0.545 (95% CI 0.532–0.557). A specific analysis of the occupational groups can be found in Table 3.

**Table 3:** Krippendorff's α for different occupational groups across the three-level response categories

Occupation	Krippendorff ‘s α	95% CI
Nursing (*n* = 14)	0.581	0.533–0.629
Medicine (*n* = 10)	0.650	0.589–0.704
Psychology (*n* = 10)	0.490	0.416–0.564
Pastoral care (*n* = 11)	0.446	0.374–0.512
Social work (*n* = 10)	0.511	0.437–0.580

#### Inter-rater reliability across the two-level response categories

The data was categorised on two levels: 1 = *No known, minimal or low risk*, 2 = *Moderate or high risk*. We applied Krippendorff's α for nominally scaled data. Across all 55 raters, Krippendorff's α was 0.511 (95% CI 0.496–0.527). A specific analysis of the occupational groups can be found in Table 4.

**Table 4:** Krippendorff's α for different occupational groups across the two-level response categories

Occupation	Krippendorff ‘s α	95% CI
Nursing (*n* = 14)	0.528	0.470–0.582
Medicine (*n* = 10)	0.580	0.500–0.656
Psychology (*n* = 10)	0.503	0.413–0.585
Pastoral care (*n* = 11)	0.370	0.284–0.449
Social work (*n* = 10)	0.523	0.445–0.601

#### Comparison of occupational groups and items in terms of mean agreement

The analysis was carried out with IBM SPSS Statistics Version 26. One Gwet’s *AC* value was available for each occupational group and item, so that a two-factor ANOVA was calculated without interaction, with the factors *item* (40 factor levels) and *occupational group* (5 factor levels). The Gwet’s *AC* values were calculated with Stata, procedure “kappaetc”. Gwet’s *AC* was calculated for each combination of occupational group*item across the 10 ratings of the relatives. The ANOVA was calculated with robust standard errors in procedure GENLIN. The multiple pairwise comparisons were corrected with Bonferroni (sequential). Table 5 shows that the items as well as the occupational groups differ highly significantly.

**Table 5:** Tests of model effects with factors item and occupational group

Source	Typ III		
	Wald-χ^2^	*df*	*p*
(Constant term)	70813.055	1	<.001
Item number	5287.033	39	<.001
Occupation	13.833	4	0.008

Dependent variable: Gwet’s *AC*

Model: (Constant term), Item number, Occupation

The descriptive statistics (Table 6) show that, on average, the nursing staff shows slightly weaker agreement than the other occupational groups. Of the pairwise comparisons, however, only the ones between nursing and medical professionals (*p* = 0.019) and between nursing and social work are significant (*p* = 0.48).

**Table 6:** Gwet’s AC and descriptive statistics of the different occupational groups across the 40 MFT items

Occupation	Mean Gwet ‘s *AC*	*N*	*SD*	Median
Nursing	0.816	40	0.171	0.878
Medicine	0.848	40	0.158	0.917
Psychology	0.837	40	0.158	0.874
Pastoral care	0.836	40	0.190	0.922
Social work	0.841	40	0.176	0.897
Total	0.836	200	0.170	0.896

## Discussion

The study aimed to adapt the Bereavement Risk Assessment Tool (BRAT; [48]) for use in German-speaking countries and regions and to test its inter-rater reliability among various healthcare professionals working in palliative care and hospice sector. The results indicate that the adapted instrument exhibits generally good to excellent inter-rater reliability for the majority of its 40 items, with 80% of the items demonstrating Gwet's *AC* values above 0.7, indicating good to almost perfect agreement. The emergence of new diagnostic criteria for PGD in ICD-11 and DSM-5-TR has created an urgent need for validated pre-death assessment tools that can reliably identify individuals at risk even before the death of a loved one.

Interestingly, in contrast to results found by Robinson et al. [46] on the BRI or Rose et al. [47] on the BRAT, inter-rater reliability decreased as the number of response categories was reduced to 3 and 2. This trend suggests that more nuanced distinctions between risk levels seemed more challenging for raters in this study to agree upon. Future research should therefore investigate whether additional training or the development of more detailed guidelines for using the MFT can improve inter-rater reliability, particularly across the different risk level categorizations. This could involve workshops or educational materials focusing on standardizing the interpretation of risk factors and achieving greater consensus in assigning risk levels. Furthermore, exploring alternative response scales or categorizations might be beneficial. It may be worthwhile to investigate whether using a different number of risk levels or employing continuous rather than categorical scales could enhance the tool's sensitivity and inter-rater reliability.

Moreover, the different findings of our study compared to results found by Robinson et al. [46] or Rose et al. [47] in reference to reduced risk levels could possibly reflect cultural differences in how grief and risk are conceptualised. This observation is particularly relevant given recent calls for culturally adapted bereavement assessments and interventions [57]. The study also revealed some noteworthy findings regarding the variability in agreement levels across different occupational groups in the palliative care and hospice sector, with nurses demonstrating slightly lower agreement compared to other professional groups. The reasons behind this discrepancy require further investigation. It is plausible that differing levels of experience with using assessment tools, variations in training, or diverse perspectives on specific risk factors could contribute to this variation. Future studies should therefore address factors contributing to the differences in inter-rater reliability between professional groups, such as the role of experience, training, and professional perspectives. This could involve qualitative studies exploring the reasoning processes underlying risk assessments made by different healthcare professionals.

The variation in agreement levels between occupational groups also persists across the different response categorizations. This finding emphasises the importance of exploring the factors underlying these differences to ensure consistent risk assessment across professional disciplines. The lower inter-rater reliability observed in certain occupational groups (e.g., pastoral care) warrants further examination. Recent research on bereavement care guidelines emphasises the need for profession-specific training and standardised assessment procedures [39]. Contemporary bereavement care models emphasize the importance of interdisciplinary collaboration and shared understanding of risk factors [58]. Raters in this study were not specifically trained using the MFT and the results showed good to very good inter-rater reliability. These results suggest that targeted training programs are likely to ensure the use of MFT in the different occupational groups. This is particularly important given the recent emphasis on compassionate communities and the expansion of bereavement care beyond traditional palliative care and hospice settings [3].

### Clinical and policy implications

The adaptation of the BRAT for German-speaking countries is particularly timely given recent developments in German palliative care guidelines. The updated S3-Leitlinie Palliativmedizin (translated: S3 guideline on palliative medicine; 2019) emphasises the importance of family-centered care and bereavement support, creating a framework within which tools like the MFT can be systematically implemented [59]. However, the current S3- Leitlinie does not provide specific guidance on bereavement risk assessment tools, representing a gap that our research helps to address.

Recent proposals for tiered bereavement care models, both internationally and in Germany (e.g. [2, 60, 61]) suggest that systematic risk assessment could facilitate more efficient allocation of limited bereavement support resources. The MFT could serve as a key component of such tiered systems, helping to identify individuals who require intensive professional support versus those who might benefit from community-based interventions.

The successful adaptation of the BRAT for German-speaking countries provides palliative care and hospice professionals with a well evaluated tool for systematic bereavement risk assessment before the death of a loved one, which could improve the identification of relatives in need of specialised support. This is particularly important given evidence that higher-risk individuals often report the most unmet needs [2, 27]. Furthermore, the tool could facilitate the implementation of evidence-based bereavement care pathways and quality improvement initiatives. Recent research emphasises the importance of structured approaches to bereavement care, including standardised assessment, care planning, and outcome monitoring [23]. Moreover, the availability of a validated German pre-death assessment tool could support research efforts to evaluate the effectiveness of different bereavement interventions and service models. This is crucial for advancing the evidence base for bereavement care and ensuring that limited resources are used effectively.

### Limitations and future research directions

While our study provides important evidence for the reliability of the MFT, several limitations should be acknowledged. First, the study was conducted using case vignettes rather than real clinical cases, which may not fully capture the complexity of actual clinical decision-making. Future research should examine the tool's performance in real-world clinical settings, potentially through prospective longitudinal studies that track bereavement outcomes [62]. Implementing the tool in real-world palliative care settings would provide valuable insights into its practicality, feasibility, and impact on patient care. This could involve randomised controlled trials examining the tool's ability to accurately identify individuals at risk for PGD and the effectiveness of subsequent interventions. By addressing these issues, future research can contribute to refining the MFT and ensuring its utility as a valuable tool for identifying and supporting individuals at risk of developing complicated grief.

Second, our sample was relatively small and may not be representative of all German-speaking healthcare professionals working in palliative care. Recruitment via existing professional networks may have introduced self-selection bias and may have limited the inclusion of professionals not embedded in such networks. As a result, the sample may not fully represent all organizations, regions, or professional perspectives within palliative and hospice care in Germany. Future studies should consider cluster-based or multicenter sampling approaches to enhance representativeness and generalizability and therefore provide more robust evidence for the tool's reliability and validity across different healthcare settings and regions.

Third, the study did not examine the tool's ability to predict actual bereavement outcomes. Future validation studies should include longitudinal follow-up to assess the tool's predictive validity for PGD according to the new ICD-11 and DSM-5-TR criteria [43]. Given the recent recognition of prolonged grief disorder as a distinct diagnostic entity, tools like the MFT are increasingly important for ensuring that bereaved individuals receive appropriate and timely support. Future research should focus on validating the tool's predictive accuracy and developing implementation strategies that maximize its clinical utility while addressing the diverse needs of different professional groups and healthcare settings.

## Conclusions

This study provides important evidence for the reliability of a German adaptation of the BRAT, addressing a significant gap in pre-death bereavement assessment tools for German-speaking countries. While the tool demonstrates good inter-rater reliability overall, variations between occupational groups highlight the importance of standardised training and implementation procedures. The availability of a validated pre-death bereavement risk assessment tool represents an important step toward implementing evidence-based, systematic approaches to bereavement care in German palliative care and hospice settings.

Overall, although the MFT does not assess deeper psychological factors such as cognitive patterns, all of which have been recognised as potential risk indicators [63], it can nonetheless be used alongside other instruments that evaluate these aspects. Although the process of assessment is multifaceted, it plays a crucial role in identifying individuals who may benefit from various types of bereavement support. Our findings show that using a bereavement risk assessment tool provides an advantage in identifying at-risk individuals compared to relying solely on clinical judgment. To what extent the instrument may serve as a pre-diagnostic tool for the development of PGD remains a desirable subject for future research.

## Data Availability

The data that support the findings of this study are available from the corresponding author upon reasonable request.
